# Effectiveness of Early Versus Late Time-Restricted Eating Combined with Physical Activity in Overweight or Obese Women

**DOI:** 10.3390/nu17010169

**Published:** 2025-01-02

**Authors:** Sarra Miladi, Tarak Driss, Ranya Ameur, Sirine C. Miladi, Samar J. Miladi, Mohamed Fadhel Najjar, Fadoua Neffati, Omar Hammouda

**Affiliations:** 1 Interdisciplinary Laboratory in Neurosciences, Physiology, and Psychology: Physical Activity, Health, and Learning (LINP2), UFR STAPS, Paris Nanterre University, 92000 Nanterre, France; sarramiladi89@gmail.com (S.M.); miledisamar.7@gmail.com (S.J.M.); 2 High Institute of Sport and Physical Education of Sfax, University of Sfax, Sfax 3000, Tunisia; ranyaamer@gmail.com (R.A.); sirinemiledi376@gmail.com (S.C.M.); 3 Research Laboratory of Evaluation and Management of Musculoskeletal System Pathologies LR20ES09, University of Sfax, Sfax 3029, Tunisia; 4 Biochemistry Laboratory, University Hospital of Monastir, Monastir 5000, Tunisia; najjar.fadhel@rns.tn (M.F.N.); neffati.fadoua72@gmail.com (F.N.); 5 Research Laboratory Molecular Bases of Human Pathology LR19ES13, Faculty of Medicine, University of Sfax, Sfax 3029, Tunisia

**Keywords:** time-restricted eating, circadian rhythms, metabolic health, physical performance

## Abstract

Aims: To evaluate the effectiveness of a dual approach involving time-restricted eating (TRE) at different times of the day combined with physical activity (PA) on functional capacity and metabolic health in overweight or obese women. Methods: Random allocation of sixty-one participants into four groups: early time-restricted eating plus physical activity (ETRE-PA, n = 15, 31.8 ± 10.76 years, 89.68 ± 13.40 kg, 33.5 ± 5.53 kg/m^2^), late time-restricted eating with physical activity (LTRE-PA, n = 15, 30.60 ± 7.94 years, 94.45 ± 15.36 kg, 34.37 ± 7.09 kg/m^2^), late time-restricted eating only (LTRE, n = 15, 27.93 ± 9.79 years, 88.32 ± 10.36 kg, 32.71 ± 5.15 kg/m^2^) and a control group (CG, n = 15, 36.25 ± 11.52 years, 89.01 ± 11.68 kg, 33.66 ± 6.18 kg/m^2^). The intervention lasted for 12 weeks in all groups. Both the ETRE-PA and LTRE-PA groups engaged in a rigorous combined aerobic and resistance-training program. Results: Significant reductions in body weight and body mass index were observed in the ETRE-PA and LTRE-PA groups compared to the CG and LTRE groups post-intervention (*p* < 0.0005). Only the ETRE-PA group exhibited a significant decrease in fat mass (*p* = 0.02), low-density lipoprotein cholesterol (*p* = 0.01), and aspartate aminotransferase (*p* = 0.002). Significant reductions in alanine aminotransferase levels were observed in the ETRE-PA (*p* = 0.004) and LTRE-PA (*p* = 0.02) groups. These two latter groups achieved higher performances in the 6-min walking test, bench press, 30-s squat, crunch test, vertical jump (*p* < 0.0005 for both), and leg extension (*p* < 0.02 for both) when compared to the LTRE and CG groups. Conclusion: The integration of TRE with PA leads to greater improvements in body composition, lipid profile, and physical performance, with no significant differences between the ETRE-PA and LTRE-PA approaches. This combined strategy offers a promising solution for overweight and obese women.

## 1. Introduction

Obesity is a major public health concern, with over 2.5 billion adults classified as overweight (body mass index, BMI ≥ 25 kg/m^2^) and 890 million as obese (BMI ≥ 30) [1]. The current rise in obesity prevalence [2] is associated with an increased risk for chronic illness [3]. Furthermore, even a small amount of body weight loss can reduce the risk of cardiovascular disease [4]. Up to now, efforts to treat and prevent overweight and obesity through dietary approaches have focused on decreasing caloric intake and enhancing the overall quality of the diet [5]. However, maintaining these strategies over time has been challenging and has led to limited success. Moreover, research has shown that irregular eating patterns can have negative effects on the body’s circadian rhythm, regardless of meal size or macronutrient composition [6]. Notably, dietary approaches that prioritize the timing of eating and length of fasting periods, known as chrono-nutrition, have been shown to enhance metabolic health irrespective of the kind, quality, or quantity of food consumed without necessarily resulting in weight loss [7]. Indeed, intermittent fasting (IF) has become a popular weight loss strategy that involves refraining from ingesting food and caloric beverages for specified times [8,9]. A specialized IF technique called time-restricted eating (TRE) involves regular fasting and eating windows that fall within a 24-h cycle and is highly recommended for persons living with obesity [10]. Earlier studies examined how TRE might affect body composition [11] and metabolic health markers [12]. The timing of daily meals in TRE can range from 4 to 12 h, with food intake periods starting either early (ETRE) or later in the day (LTRE). To date, few studies have compared ETRE and LTRE outcomes in terms of metabolic health and fitness [13]. Despite the potential effects of exercise on physical fitness and physiological adaptation during fasting, only a few studies have investigated the combined effect of physical activity and TRE in metabolic disorders [14]. A review by Albosta et al. [15] concluded that endurance exercise practiced in a fasted state induced a greater improvement in insulin sensitivity in patients with type 2 diabetes than exercise in the fed state. Furthermore, a meta-analysis conducted by Vieira et al. [16] showed that the combination of TRE and aerobic exercise induced greater body weight loss, a decrease in liver genes related to lipogenesis, and an improvement in fatty acid oxidation compared to the control group. However, previous studies highlighted metabolic differences between early and late TRE schedules [17]. Research has indicated variability in how each approach impacts circadian rhythms and glucose metabolism, especially concerning breakfast consumption and the timing of exercise relative to the last meal [18]. ETRE, which restricts food intake to the morning and early afternoon, aligns more closely with the body’s central and peripheral circadian clock [19]. This synchronization is believed to positively influence metabolic regulation, as it encourages eating when metabolic processes such as glucose metabolism and insulin sensitivity are most active. ETRE may, therefore, facilitate greater reliance on fat oxidation, as the extended fasting period that follows encourages the liver to break down fatty acids into ketone bodies to supply energy to vital organs and tissues. This metabolic shift could lead to improvements in glucose regulation, body weight, and other metabolic markers (decreased blood pressure and LDL-c levels) [20]. In contrast, LTRE, where food intake is delayed until later in the day, may disrupt circadian alignment by extending the eating period into the evening, when metabolic activity is lower. Studies have suggested that LTRE may impair glucose and insulin sensitivity, because eating later in the day interferes with the natural decline in metabolic efficiency that typically occurs in the evening. [21]. Furthermore, previous studies have indicated that TRE is associated with elevated cholesterol levels [22]. This alteration can be explained by muscle loss.

Moreover, the timing of exercise has a profound impact on metabolic responses. Exercising in a fasted state promotes fat oxidation and enhances metabolic flexibility, while postprandial exercise relies more on carbohydrate stores and may affect glucose dynamics after activity [23]. These aspects highlight the importance of adapting TRE to individual health objectives and chronobiological needs.

Additionally, a study by Batitsuci et al. [24] demonstrated that an 8-week intervention of IF combined with high-intensity interval training (HIIT) resulted in significant weight reduction, increased muscle mass, and enhanced physical performance.

Investigating the effects of ETRE vs. LTRE in combination with PA could provide valuable insights into how the eating window influences the effectiveness of TRE. Such a comparison may clarify whether ETRE with PA provides superior health benefits or whether the same intervention can help mitigate potential metabolic drawbacks associated with LTRE. The findings from this research could play a crucial role in optimizing TRE interventions, especially for enhancing body composition and cardiometabolic health outcomes in patients.

Thus, the aim of the present study was to investigate the effect of concurrent (aerobic and resistance) training in combination with ETRE or LTRE on body composition, metabolic profile, and functional capacity in overweight or obese women.

## 2. Materials and Methods

### 2.1. Participants

**Figure 1:**
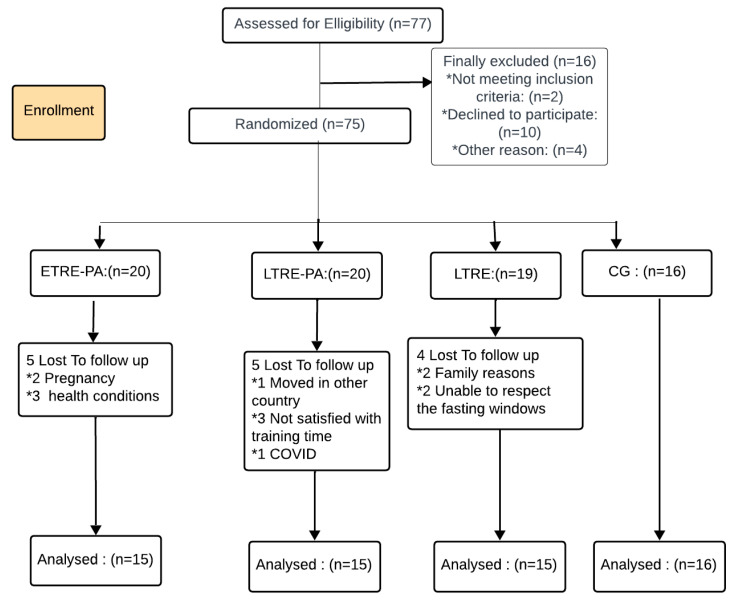
Flowchart of participants’ recruitment. ETRE-PA: Early time-restricted eating combined with physical activity group; LTRE-PA: Late time-restricted eating combined with physical activity group; LTRE: Late time-restricted eating group; CG: Control group.

The required sample size was determined a priori using G*Power software 3.1.9.7, following the procedures recommended by Beck [25] and based on a previous study employing the same paradigm. Statistical power was estimated at 0.85, with an alpha level of 0.05 [26]. A minimum of 40 participants were deemed necessary. This rigorous approach in determining the sample size helps ensure that the study has sufficient statistical power to detect meaningful effects and minimize the risk of incurring a type 2 statistical error. Initially, seventy-seven women volunteered to participate in the present study. Before agreeing to participate, each participant was provided with a detailed description of the protocol as well as an explanation of the potential risks and benefits involved. All participants provided written informed consent before participating in the study. The inclusion criteria required participants to have a BMI greater than 25 kg/m^2^, be able to attend laboratory assessment and training sessions, be free from any known cardiovascular disease, and not be pregnant. Despite an attrition rate of 21% of participants in the study), the study successfully enrolled 61 sedentary women who were overweight or obese (Figure 1). Participants’ body composition was assessed using bioelectrical impedance (Model TBF-300, Tanita Corp, Tokyo, Japan) [27]. The study design was approved by the local ethics committee (CPP SUD, n° 0460/2022, Sfax, Tunisia, approval date: 16 December 2022) and was registered in the Pan African Clinical Trials Registry PACTR202301473691345 and conducted in accordance with the Helsinki guidelines.

### 2.2. Study Design

**Figure 2:**
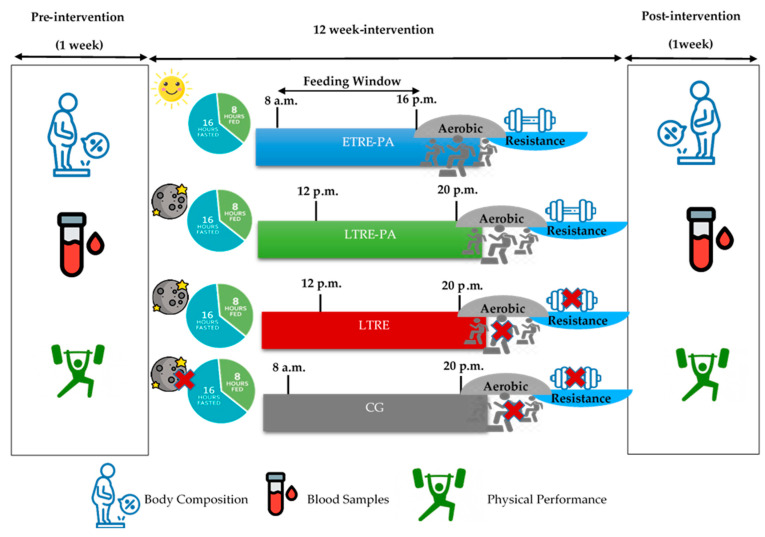
Study Design. ETRE-PA: Early time-restricted eating combined with physical activity; LTRE-PA: Late time-restricted eating combined with physical activity; LTRE: Late time-restricted eating; CG: Control group.

Participants were randomly assigned to one of four groups: ETRE-PA (n = 15, 31.8 ± 10.76 years, 89.68 ± 13.40 kg, 33.5 ± 5.53 kg/m^2^), LTRE-PA (n = 15, 30.60 ± 7.94 years, 94.45 ± 15.36 kg, 34.37 ± 7.09 kg/m^2^), LTRE (n = 15, 27.93 ± 9.79 years, 88.32 ± 10.36 kg, 32.71 ± 5.15 kg/m^2^) and control group (CG) (n = 16, 36.25 ± 11.52 years, 89.01 ± 11.68 kg, 33.66 ± 6.18 kg/m^2^). The two experimental groups adhered to the TRE protocol ad libitum and participated in training sessions that combined aerobic exercises with muscle-strengthening activities (Figure 2). The intervention spanned twelve weeks, during which body composition, biochemical, and physical performance were measured at the same time for all groups. The participants in the CG were advised to maintain their regular daily meals and bedtime routines throughout the study. To ensure consistency, all measurements were taken between 7:00 am and 12:00 pm after a 16-h fast. The three intervention groups (ETRE-PA, LTRE-PA, and LTRE) received dietary guidelines with specific recommendations on meal timing, daily caloric intake, and macronutrient distribution designed to achieve a total daily intake of 1200–1500 kcal. Participants were instructed to consume three daily meals without additional snacks, all within 8 h period followed by a 16 h fasting period. However, a 4-h difference was established between the meal timing of the ETRE-PA, LTRE-PA, and LTRE groups. Participants in the ETRE-PA group were required to abstain from consuming any calories between 4:00 pm and 8:00 am the following day throughout the intervention. In contrast, participants in the LTRE-PA and LTRE groups were instructed to follow their customary dietary patterns but within a designated eating window from 12:00 pm to 8:00 pm, allowing an 8-h period for food consumption while restricting intake outside this timeframe. The 4-h difference between the ETRE and LTRE groups was chosen based on the established principles of circadian physiology. This distinction allowed for a meaningful comparison of the effects of food intake during different time windows on participants’ metabolic and hormonal rhythms. ETRE aligns food intake with the peak activity of metabolic processes, such as insulin sensitivity and energy expenditure, which are known to follow a diurnal pattern [28]. Conversely, LTRE occurs closer to the biological evening, a period associated with reduced metabolic efficiency and increased susceptibility to weight gain and glucose intolerance [29]. This time window distinction is particularly relevant, as previous studies have demonstrated that consuming meals earlier in the day can lead to better weight management, improved glucose control, and enhanced lipid profiles compared to late eating patterns [30,31]. By maintaining a 4-h difference, we aimed to ensure a clear contrast in the alignment of food intake with circadian rhythms, which is crucial for investigating the temporal effects of eating patterns on health outcomes.

### 2.3. Training Sessions

ETRE-PA and LTRE-PA participants were required to attend all training sessions at the fitness center, where they engaged in a 70-min workout three times a week. Each training session consisted of a comprehensive routine, including warm-up (5 min), aerobic exercise (30 min), strength training (30 min), and stretching and breathing exercises (5 min).

#### 2.3.1. Endurance Training

The training program included auditory stimulation and intensity modulation (tempo). Sessions were scheduled three times per week on Monday, Wednesday, and Friday at the end of the feeding window for each group (i.e., at 4:00 pm for ETRE-PA and 8:00 pm for LTRE-PA). The intervention was divided into four phases, with a progressive increase in intensity within each phase lasting three weeks (nine training sessions). In the first phase, participants exercised for 30 min at 50–60% of their estimated maximum heart rate (HRmax: 220-age) with an auditory tempo of 135–140 beats per minute (BPM). The second phase involved exercising for 30 min at 55–65% of HRmax at the same tempo. In the third phase, the participants exercised for 30 min at an intensity of 60–70% HRmax with a faster auditory tempo of 140–152 BPM. Finally, the fourth phase required participants to exercise for 60 min at an intensity of 65–75% of HRmax, with the same tempo as in phase three.

#### 2.3.2. Strength Training

The training was structured as circuit weight training, incorporating handheld weights, weight machines (Technogym S.P.A., Cesena, Italia), and participants’ own body weight. Each participant engaged in two preliminary training sessions to familiarize themselves with the equipment and master the correct techniques for executing the exercises [32].

### 2.4. Outcome Measures

The study spanned a period of 14 weeks, with the 12-week intervention starting after one week of baseline measures. Throughout this period, all participants maintained their usual dietary intake. Outcome measures were measured both at baseline and post-intervention. Body composition was assessed by measuring total body mass and composition, including lean mass, fat-free mass (FFM), bone mass, and total water (TBW), before and after the intervention period using bioelectrical impedance analysis (Model TBF-300, Tanita Corp, Tokyo, Japan). Measurements were taken in the morning between 7:00 and 10:00 am following overnight fasting, with participants dressed simply and without shoes or socks. A wall-mounted stadiometer was used to measure the participants’ standing height to the nearest 0.1 cm.

### 2.5. Blood Samples

Blood samples were obtained from the antecubital vein after an overnight fasting 16-h fasting period, between 7:00 and 10:00 am, both three days before and after the intervention. Participants were advised to maintain their regular diet and avoid vigorous PA within 48 h prior to each blood sampling session. The blood samples were immediately placed in an ice bath to prevent degradation and then centrifuged at 2500 rpm for 10 min at 4 °C. The resulting plasma was divided into smaller aliquots to avoid multiple freeze-thaw cycles and stored at −80 °C for 2 weeks until analysis. All measurements were performed using a Cobas 6000™ machine from Roche Diagnostics Manheim (Roche Diagnostics GmbH, Manheim, Germany). The lipid profile variables, including total cholesterol (TC) and triglycerides (TG), were assessed using an enzymatic colorimetric technique. High-density lipoprotein cholesterol (HDL-c) was determined using a direct enzymatic colorimetric method, while low-density lipoprotein cholesterol (LDL-c) levels were calculated using the Friedewald formula [33]:

[LDL] = [TC] − ([HDL] + [TG/2.2])

For the glycemic profile, fasting blood glucose was measured using an enzymatic hexokinase assay, and insulin levels were assessed using an electrochemical luminescence-based assay. Insulin resistance was quantified using the homeostatic model assessment (HOMA-IR), calculated as follows: [insulinemia (U/mL) × glycemia (mmol/L)]/22.5. Aspartate aminotransferase (ASAT) and alanine aminotransferase (ALAT) levels were determined using a UV kinetic assay to assess liver function. Total Bilirubin levels were measured using a colorimetric technique. Creatinine was assessed using an enzymatic method. Alkaline phosphatase levels were assessed using a visible kinetic assay, which measures enzyme activity at a wavelength of 405 nm. This method involves monitoring the rate of reaction over time as the substrate is converted into a product, allowing for the quantification of alkaline phosphatase activity in the samples. This measurement is crucial for assessing bone and liver health. Finally, C-reactive protein (CRP) levels were measured using the immuno-turbidimetric method. All measurements were conducted within a single assay run by the same investigator.

### 2.6. Functional Capacity

All functional capacity tests were conducted between 10:30 a.m. and 12:00 p.m., as detailed in the following section.

#### 2.6.1. Six-Minute Walk Test (6MWT)

The 6-min walk test (6MWT) was selected due to its well-established validity and reliability as an effective measure of functional capacity and cardiovascular fitness. It is widely recognized and utilized in both clinical and research settings to evaluate endurance and overall physical function, particularly in populations with obesity. The test provides valuable insights into an individual’s aerobic capacity, functional endurance, and overall mobility, making it a suitable choice for assessing the impact of interventions on physical health in our study population [34]. During this test, participants were instructed to walk along a 30-m corridor at their maximum speed for six minutes. The corridor was marked every 5 m with colored tape to allow precise measurement of the distance covered (6 MWD). Before the test began, participants rested for about 10 min on a chair located near the starting point. Blood pressure was measured during this period to establish the baseline value. Once the participants began walking, the stopwatch was started, and each completed lap was recorded when the participants returned to the starting point. In addition to the completed laps, any extra distance covered beyond the 30-m corridor was also recorded using the nearest colored tape marker on the floor. This procedure ensured the collection of accurate and reliable data during the test.

#### 2.6.2. Strength Tests

For strength assessments, we recorded the one repetition maximum (1-RM) for both the bench press (BP) and leg press (LE) exercises. Each 1-RM assessment was preceded by a specific warm-up routine, which involved performing five repetitions with a weight typically manageable for ten repetitions, following the techniques outlined in previous studies [35,36]. The 1-RM measurements were conducted at the beginning of the study and after 12 training sessions for all prescribed exercises. This allowed us to make the necessary adjustments to account for any potential increases in strength that may have occurred during the course of the training program. To estimate 1-RM values, we employed the Epley method, which calculates the 1-RM as follows: 1-RM = Weight Lifted × (1 + 0.0333 × Number of Repetitions) [35].

#### 2.6.3. Vertical Jump Test

To assess lower body explosiveness, a Takei Vertical Jump Meter (Takei, Niigata, Japan) was used. Three maximum attempts were measured with a 30-s rest time between jumps. The participants were encouraged throughout the testing process. The highest jump was selected for additional analysis.

#### 2.6.4. The 30-s Crunch and Squat Tests

**Figure 3:**
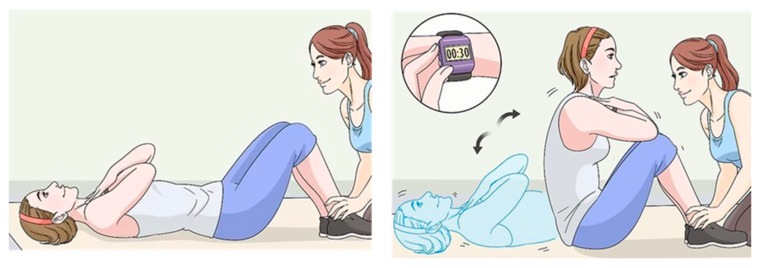
Crunch test.

The 30-s Crunch and Squat Tests is a simple fitness assessment designed to evaluate the strength and endurance of the abdominal muscles. To conduct the test, the participant lies on a mat with knees bent at a 90-degree angle and feet flat on the floor. The arms are crossed over the chest. The test begins by engaging the core muscles and lifting the shoulder blades off the ground, raising the upper body toward the knees until the elbows touch the thighs or reach a 30-degree angle. The movement is then reversed to return to the starting position. This action is repeated continuously and rhythmically for 30 s, with each complete crunch being counted (Figure 3).

**Figure 4:**
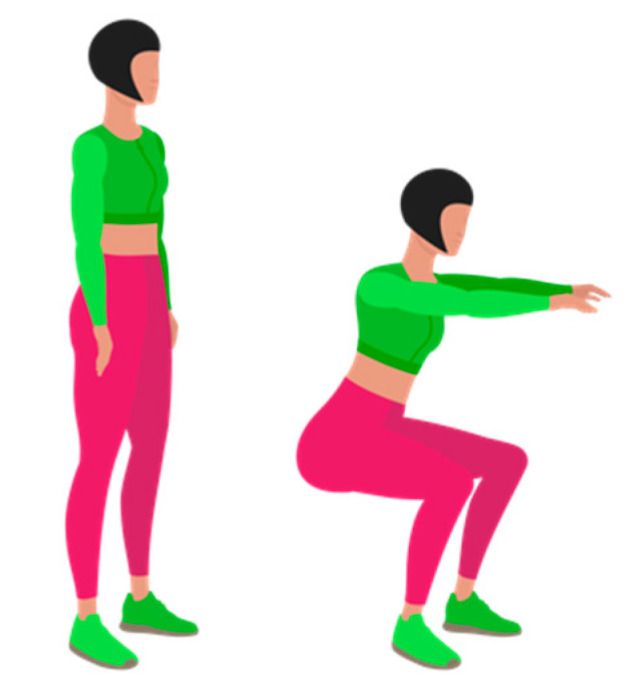
Squat test.

The squat test was also used to assess lower body strength and endurance. Participants were instructed to stand with their feet shoulder-width apart, engage their core, bend their knees, and lower their hips toward the ground. It is important to maintain proper form by keeping the back straight and knees in line with the toes. Once in a seated position, participants must stand back up by pushing through their heels and engaging their glutes and thigh muscles. The test lasts for 30 s, during which participants should aim to complete as many correctly executed squats as possible (Figure 4).

## 3. Statistical Analysis

Statistical analyses were conducted using SPSS software (v.23, IBM, New York, NY, USA). All data are presented as the mean ± standard deviation (SD). Parametric tests were performed after confirming the assumption of normality using the Kolmogorov–Smirnov test. A two-way ANOVA for repeated measures was used to evaluate differences across groups (4 groups (ETRE-PA, LTRE-PA, LTRE, CG) × Two measurement points (before and after the intervention)). This analysis was applied to assess changes in body composition parameters (body weight, BMI, FM, FFM, MM, and TBW), cardiometabolic parameters (Glycemic profile (Fasting Glycemia, Fasting insulin, Insulin Resistance), hepatic profile ASAT, ALAT, Gamma GT, TB, creatinine) lipid profile (LDL-c, HDL-c, TC) and TG. CRP and Cortisol levels), 6MW distance (6MWD), 1-RM for extension leg (1-RM EL), 1-RM for bench press (1-RM BP), squat performance, and crunch performance. ANOVA effect sizes were calculated using partial eta squared (ηp^2^) to assess the magnitude of the observed effects. When significant main or interaction effects were observed, pairwise comparisons were performed using the Bonferroni post hoc test. The significance level for all statistical procedures was set at *p* < 0.05.

## 4. Results

### 4.1. Body Composition

Two-way ANOVA for repeated measures showed significant changes over time for body weight (F_(1, 57)_ = 127.41, *p* < 0.0005, ɳp^2^ = 0.69), BMI (F_(1, 57)_ = 76.61, *p* < 0.0005, ɳp^2^ = 0.57), FM (F_(1, 57)_ = 8.16, *p* = 0.006, ɳp^2^ = 0.12) and LM (F_(1, 57)_ = 4.29, *p* = 0.04, ɳp^2^ = 0.07). A group effect was observed only for FM (F_(3, 57)_ = 3.01, *p* = 0.03, ɳp^2^ = 0.13). A (Group × Time) interaction was identified for weight (F_(3, 57)_ = 30.88, *p* < 0.0005, ɳp^2^ = 0.61), BMI (F_(3, 57)_ = 15.62, *p* < 0.0005, ɳp^2^ = 0.57) and LM (F_(3, 57)_ = 3.08, *p* = 0.03, ɳp^2^ = 0.14). The Bonferroni post hoc analysis revealed a significant decrease in body weight for both ETRE-PA (*p* < 0.0005, ∆ (%) = −10.98) and LTRE-PA (*p* < 0.0005, ∆ (%) = −7.99) groups after the intervention compared to before the intervention. Furthermore, during the post-intervention period, these decreases were also significant in the ETRE-PA and LTRE-PA groups compared to the CG and LTRE groups (*p* < 0.0005). Additionally, a significant reduction in BMI was observed in both the ETRE-PA (*p* < 0.0005, ∆ (%) = −12.06) and LTRE-PA (*p* < 0.0005, ∆ (%) = −8.39) groups after the intervention. However, post-intervention, only the ETRE-PA group showed a significantly lower BMI compared to the CG group (*p* = 0.02). A significant decrease in FM (*p* = 0.02, ∆ (%) = −7.44) and LM (*p* = 0.01, ∆ (%) = −7.32) was observed in ETRE-PA only after the intervention. No significant difference was observed between the groups concerning TBW.

### 4.2. Metabolic Parameters

ANOVA for repeated measures showed a significant time effect for TC, LDL-c, ALAT, ASAT, and alkaline phosphatase (Table 1). Furthermore, the group effect was observed only for alkaline phosphatase (F_(3, 57)_ = 7.33, *p* < 0.0005, ɳp^2^ = 0.27). Additionally, the interaction effect (Group × Time) was observed only for TC (F _(3, 57)_ = 3.16, *p* < 0.0005, ɳp^2^ = 0.14). When comparing before to after the intervention, the Bonferroni post hoc analysis showed a significant reduction in ALAT levels in both the ETRE-PA (*p* = 0.004, ∆ (%) = −47.75) and LTRE-PA (*p* = 0.02, ∆ (%) = −37.58) groups. Only the ETRE-PA group showed a greater decrease in TC (*p* = 0.001, ∆ (%) = −14.58) and LDL-c (*p* = 0.01, ∆ (%) = −14.29). Conversely, only the LTRE-PA group exhibited a reduction in ASAT levels (*p* = 0.02, ∆ (%) = −24.61). Furthermore, when comparing between groups, a significant difference was observed in alkaline phosphatase between ETRE-PA and CG groups (*p* = 0.002) and LTRE-PA and CG (*p* < 0.005) groups. Glucoregulatory factors, HDL-c, Gamma GT, cortisol, bilirubin, creatinine, CRP, and TSH levels remained unaffected by either intervention (*p* > 0.05).

### 4.3. Functional Capacity

#### 4.3.1. 6MWT

Two-way ANOVA for repeated measures revealed a significant effect of time (F_(1, 57)_ = 76.29, *p* < 0.0005, ηp^2^ = 0.57), group (F_(3, 57)_ = 22.29, *p* < 0.0005, ηp^2^ = 0.54), and (Group × Time) interaction (F_(3, 57)_ = 23.43, *p* < 0.0005, ηp^2^ = 0.55). Bonferroni post hoc analysis showed higher performances in both ETRE-PA and LTRE-PA when comparing after-intervention to before-intervention (Figure 4). When comparing the groups, a significantly higher 6 MWD performance was observed in the ETRE-PA and LTRE-PA groups compared to the CG group (*p* < 0.0005).

#### 4.3.2. LE and BP 1-RM

Two-way ANOVA showed significant effects of time on both LE 1-RM (F_(1, 57)_ = 29.67, *p* < 0.0005, ηp^2^ = 0.31) and BP 1-RM (F_(1, 57)_ = 12.94, *p* = 0.001, ηp^2^ = 0.18). Additionally, group effects (F_(3, 57)_ = 14.67, *p* < 0.0005, ηp^2^ = 0.43; F_(3, 57)_ = 11.02, *p* < 0,0005, ηp^2^ = 0.99, respectively) were observed for the same parameters. However, interaction effects were revealed only for LE 1-RM (F_(3, 57)_ = 9.68, *p* < 0.0005, ηp^2^ = 0.33). The Bonferroni post hoc analysis showed significantly greater improvements in LE 1-RM in the ETRE-PA and LTRE-PA groups compared to the LTRE (*p* = 0.001, *p* < 0.0005, respectively) and CG groups (*p* < 0.0005). Moreover, when comparing before- to post-intervention, both ETRE-PA and LTRE-PA demonstrated enhanced performance in LE 1-RM (*p* < 0.0005) and BP 1-RM (*p* < 0.005).

#### 4.3.3. Explosiveness and Endurance Strength

**Figure 5:**
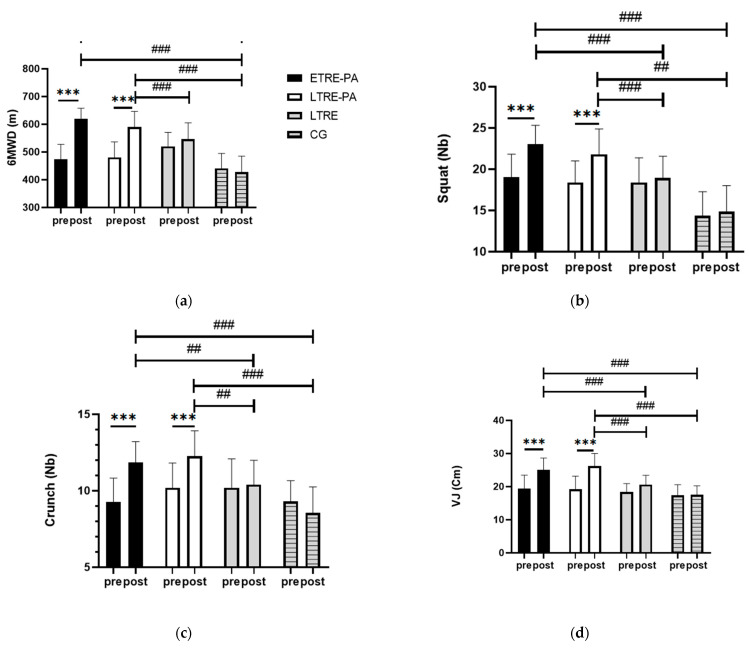
Physical performance indices between groups. ETRE-PA: Early time-restricted eating combined with physical activity; LTRE-PA: Late time-restricted eating combined with physical activity; LTRE: Late time-restricted eating; CG: Control Group; PRE: Before the intervention; POST: After the intervention; VJ: Vertical jump test; (**a**): 6 min walking test; (**b**): Squat test; (**c**): Crunch test; (**d**) Vertical jump test. *** *p* < 0.0005; ## *p* < 0.01; ### *p* < 0.0005.

Two-way ANOVA for repeated measures revealed a significant effect of time in the vertical jump test (F_(1, 57)_ = 91.19, *p* = 0.001, ηp^2^ = 0.61), 30-s squat test (F_(1, 57)_ = 45.24, *p* < 0.0005, ηp^2^ = 0.44), and 30-s crunch test (F_(1, 57)_ = 24.54, *p* < 0.0005, ηp^2^ = 0.30). A group effect was observed in the vertical jump test (F_(3, 57)_ = 9.95, *p* < 0.0005, ηp^2^ = 0.34), 30-s squat test (F_(3, 57)_ = 19.05, *p* < 0.0005, ηp^2^ = 0.50), and 30-s crunch test (F_(3, 57)_ = 7.12, *p* < 0.0005, ηp^2^ = 0.27). Furthermore, an interaction (Group × time) effect was observed for these parameters (F_(3, 57)_ = 16.28, *p* < 0.0005, ηp^2^ = 0.46; F_(3, 57)_ = 8.57, *p* < 0.0005, ηp^2^ = 0.31; F_(3, 57)_ = 14.45, *p* < 0.0005; ηp^2^ = 0.43, respectively). The Bonferroni post hoc analysis showed higher performance in the vertical jump test, 30-s squat test, and 30-s crunch when comparing before- to after-intervention (Figure 5a,b). Both the ETRE-PA and LTRE-PA groups exhibited improvements in the vertical jump test when compared to the CG (*p* < 0.0005) and LTRE (*p* < 0.0005). In the squat test, both ETRE-PA and LTRE-PA groups exhibited higher improvements when compared to the CG (*p* < 0.0005) and LTRE groups. (*p* < 0.0005, *p* = 0.008, respectively) Additionally, in the crunch test, both the ETRE-PA and LTRE-PA groups exhibited greater improvements compared to CG (*p* < 0.0005) and LTRE (*p* = 0.01, *p* = 0.003, respectively) (Figure 5c).

## 5. Discussion

The present study investigated for the first time the impact of ETRE and LTRE combined with PA compared to LTRE and CG on body composition, cardiometabolic health, and physical performance among women with overweight or obesity. The main findings revealed significant reductions in body weight and BMI in both the ETRE-PA and LTRE-PA groups. Specifically, the ETRE-PA group showed a reduction in FM, FFM, LDL-c, and ALAT levels. Conversely, the LTRE-PA group showed reduced ALAT and ASAT levels. However, the glycemic profile and cortisol, bilirubin, creatinine, CRP, and TSH levels were not affected by either intervention. Regarding physical performance, the ETRE-PA and LTRE-PA groups showed significant improvements in all tests compared to the LTRE and CG. Previous studies have established TRE as an effective lifestyle intervention to improve body composition and overall health [36,37], even without caloric counting [38]. The same authors showed that limiting the eating window to 8 h per day without any caloric monitoring led to an average weight loss of 4.6 kg The impact of the timing of food intake ETRE vs. LTRE on body composition has gained considerable interest in recent years [39]. Few studies have compared the effects of ETRE and LTRE combined with physical exercise on body composition and health parameters. The existing literature presents mixed results, mostly without notable differences [40,41], which aligns with the results of our study.

Queiroz et al. [42] compared the effects of ETRE (8:00 a.m. to 4:00 p.m.), LTRE (12:00 p.m. to 8:00 p.m.) and a control condition (8:00 a.m. to 8:00 p.m.) in overweight and obese adults. Significant decreases in FFM and fat mass were observed in all three groups, without notable differences between them. Additionally, a 14-week study by Jamshed et al. [43] compared ETRE (7:00 a.m. to 3:00 p.m.) with a control group (eating window ≥ 12 h). The ETRE group showed greater weight loss. A single long-term trial (12 months) presented by Liu et al. [44] compared ETRE (8 a.m. to 4 p.m.) with a CG on a hypocaloric diet (1500-1800 kcal/day for men, 1200–1500 kcal/day for women) in overweight and obese adults. After 12 months, reductions in fat mass (5.9 and 4.5 kg, respectively) and lean mass (1.7 and 1.4 kg, respectively) were statistically significant in both the ETRE and control groups, with no notable differences between groups. Similarly, our study showed a significant decrease in lean weight in the ETRE-PA group when comparing pre-and post-intervention values. The decrease in lean mass may be explained by insufficient protein intake [45]. Other mechanisms, such as the disruption of anabolic hormones like growth hormone (GH), may also play a role. An alteration in GH secretion can disrupt protein synthesis, leading to a reduction in lean mass [46]. Additionally, the insufficiency of ketone bodies as an energy source may contribute to the degradation of skeletal muscles [47]. Furthermore, the present study demonstrated that ETRE-PA led to a selective loss of fat mass when comparing pre-and post-intervention results, with no significant difference when comparing between groups. These results are consistent with those of Low et al. [48], who also indicated that ETRE is an effective intervention for reducing fat mass. This effectiveness could be attributed to the alignment of the body’s circadian rhythm with ETRE, which has been shown to improve metabolic processes and reduce the risk of metabolic disorders, as reported by Patterson et al. [49]. On the other hand, Kotarsky et al. [50] showed that 8 weeks of TRE combined with training compared to control condition reduced body weight (−3.3% vs. −0.2%) as well as fat mass (−9.0% vs. −3.3%) and increased lean mass percentage (+2.4% vs. +1.2%). According to Boyd et al. [51], these improvements may be attributed to a reduction in energy intake throughout the study. Indeed, it has been shown that the type of diet plays a crucial role in the effects of TRE on health oy. Fasting has been proven to stimulate fat metabolism and promote the metabolic transition from glucose oxidation to fat oxidation when glycogen stores are depleted [52,53]. This can be accompanied by increased lipolysis of adipose tissue and the release of free fatty acids and glycerol into the plasma [54,55]. Mobilization and use of fatty acids in adipocytes increases caloric expenditure, which can prevent obesity [56]. Regarding cardiometabolic health, previous studies [57,58] have shown that late eating is associated with poorer cardiometabolic health. The circadian misalignment could explain these results [59]. Additionally, glucose tolerance is better in the biological morning, which seems to be related to diurnal variations in β-cell response, peripheral insulin sensitivity, insulin clearance, and glucose efficiency [60,61]. Fatty acid oxidation in skeletal muscles and the thermic effect of food are also higher in the morning. This suggests that it is optimal to consume the majority of daily calories earlier in the day while reserving the late afternoon and the night for fasting and sleep [62]. In the present study, our results showed that the combination of LTRE and physical exercise had no negative effects on cardiometabolic health. In contrast, we observed that the combination of physical training and LTRE resulted in significant improvements in markers of liver damage (ASAT, ALAT, and alkaline phosphatase). The combination of physical training and LTRE led to significant improvements in the markers of liver function and damage, including ASAT and alkaline phosphatase. These benefits can be attributed to the characteristic fasting period of LTRE, which supports toxin clearance and facilitates liver regeneration. Moreover, the timing of nutrient intake in LTRE-PA, occurring closer to the resting period, provides the liver with sufficient time to efficiently metabolize and store nutrients after exercise. This strategic synchronization between exercise, fasting, and feeding reduces the liver’s metabolic workload during the subsequent fasting phase, contributing to improved liver function and reduced hepatic stress. This process occurs independently of changes in lipid metabolism, which may take longer to show improvements in lipoprotein profiles like LDL-c and TC [63]. Additionally, the ETRE-PA group showed a significant improvement in lipid profile (TC and LDL-c) as well as liver markers (ALAT and alkaline phosphatase), suggesting a synergistic effect of the combination of both interventions. Furthermore, these enhancements may be explained by a reduction in systemic inflammation and oxidative stress, which are key contributors to liver damage and dyslipidemia. The enhancement of the lipid profile observed exclusively in the ETRE-PA group can be attributed to the synergistic effects of circadian synchronization, physical activity, and prolonged fasting. Early feeding aligns with the liver’s peak lipid metabolism activity, facilitating efficient clearance of LDL-c and other lipids. Physical activity further amplifies lipid turnover and oxidation, while an extended fasting period post-feeding enhances fat utilization. The observed reduction in ASAT indicates improved muscle recovery and metabolic efficiency, suggesting that the benefits of ETRE-PA are driven by circadian alignment and exercise-enhanced lipid metabolism rather than a reduction in hepatic stress, as evidenced by the unchanged ALAT levels [64]. Additionally, these results are consistent with a meta-analysis by Dai et al. [65], which reported a significant improvement in the lipid profile after combining TRE and physical exercise. Furthermore, in contrast to our findings, another study conducted by Real-Hohn et al. [66] showed a significant decrease in insulin, improved oxidative stress biomarkers, better glucose tolerance, and hexokinase activity in the liver, heart, and skeletal muscles after intermittent fasting combined with high interval training group compared to the control group. It is important to note that the improvements observed in lipid profiles and liver enzymes in our study were independent of factors regulating blood glucose, cortisol, CRP, and TSH levels, suggesting that TRE may have direct effects on lipid metabolism and liver function. However, we also observed a trend toward a reduction in blood glucose regulation parameters, including insulin sensitivity, fasting insulin, and blood glucose levels in the ETRE-PA and LTRE-PA groups after the intervention. Perhaps an increase in exercise intensity could further improve these parameters. According to our results, another study by Wei et al. [67] showed no change in the glycemic profile during similar intermittent fasting. The same researchers explained this stagnation by the fact that the baseline levels of the participants were below 9.9 mmol/L. Therefore, they suggested that an improvement in fasting blood glucose levels might be observable beyond these values. One of the main objectives of the present study was to highlight the effectiveness of TRE and PA in improving various aspects of physical performance. To our knowledge, this study is the first to examine the combined effects of different TRE times during the day and physical activity on physical performance in overweight and obese women by evaluating exercise capacity in various intervention groups and comparing them to a control group. Our results showed that a 16/8 TRE regimen followed for 12 weeks resulted in a significant improvement in walking distance during the 6 MWT in the ETRE-PA and LTRE-PA groups. Most previous studies [68,69,70,71,72] have evaluated the effect of fasting combined with physical exercise on athletes and have produced mixed results. During Ramadan (14/10), negative effects on performance appear likely due to factors such as disrupted sleep and hydration. These effects were more pronounced in elite athletes, but among amateur athletes, the difference is minimal [70,72]. Conversely, no significant differences were observed after TRE (16/8) in aerobic [73,74,75,76], anaerobic [71,76], as well as strength and power [68,69,74,76,77,78]. However, a meta-analysis by Zouhal et al. [79] mentioned that fasting training in sedentary and untrained subjects could promote physiological muscle adaptations, potentially leading to improved endurance performance. Furthermore, the enhanced performance observed in the ETRE-PA and LTRE-PA groups can be attributed to the synergistic effects of PA and TRE. Physical activity serves as a primary stimulus for improving explosive power and muscular strength/endurance [80], while TRE contributes to enhanced recovery, energy utilization, and metabolic efficiency. In the ETRE-PA group, the alignment of feeding with circadian rhythms likely maximized the nutrient availability for muscle repair, energy restoration, and performance boosting. In the LTRE-PA group, prolonged fasting may have promoted metabolic flexibility and mitochondrial efficiency, supporting comparable performance improvements. These findings highlight that while both TRE protocols enhance performance, the underlying mechanisms may vary based on the timing of nutrient intake relative to the circadian rhythm and physical activity.

### Limitations

This study has some limitations. First, it remains unclear whether the metabolic health benefits associated with TRE are due to a shortened eating window, a potential reduction in energy intake, or a combination of both, as neither caloric intake nor protein consumption was assessed across the groups during the intervention. Moreover, the intensity of muscle-strengthening exercises may have been insufficient to prevent the loss of lean mass. Additionally, the longitudinal design of the study, while valuable, did not allow for the control or monitoring of menstrual cycle phases, further complicating the interpretation of results related to these factors. Lastly, future studies could consider incorporating an additional group that engages solely in physical activity to determine whether the observed benefits can be attributed exclusively to the exercise intervention.

## 6. Conclusions

Our findings suggest that the combination of TRE and PA positively affects body composition, cardiometabolic health, and functional capacity. Both the ETRE-PA and LTRE-PA interventions contributed to improved patient outcomes, with exercise reducing some of the adverse effects of LTRE on body composition and cardiometabolic health in patients reported in the literature. However, no significant effects were observed on some metabolic parameters related to glycemic variables, indicating that maintaining a relatively short eating window may lead to better outcomes. Adapting these interventions to individual characteristics, such as chronotype, could further optimize outcomes by aligning eating windows and physical activity with participants’ natural circadian rhythms. This personalized approach may enhance the effectiveness of both TRE (ETRE and LTRE) with PA interventions. Additionally, increasing the intensity of exercise in the fasted state, particularly in resistance training, could lead to more significant improvements in body composition, especially in terms of increasing lean mass and improving metabolic health.

## Figures and Tables

**Table 1:** Biochemical parameters before and after the intervention.

Biomarkers	ETRE-PA		LTRE-PA		LTRE		CG		ANOVA		
	Pre	Post	Pre	Post	Pre	Post	Pre	Post	F_(1, 57)_	P (Time)	ηp^2^ (Time)
**LDL-c (mmol/L)**	2.75 ± 0.76	2.32 ** ± 0.54	2.65 ± 0.76	2.46 ± 0.95	2.38 ± 0.72	2.12 ± 0.54	3.17 ± 1.08	2.39 ± 0.63	24.27	0.000	0.29
**TC (mmol/L)**	4.47 ± 0.90	3.76 *** ± 0.64	4.29 ± 0.96	3.98.96 ± 1.38	4.13 ± 0.91	3.69 ** ± 0.69	4.96 ± 1.24	3.87 ± 0.85	43.07	0.000	0.43
**ALAT (UI/L)**	14.73 ± 10	6.38 ** ± 2.14	13.60 ± 7.28	7.04 * ± 3.34	12.66 ± 10.8	7.80 ± 2.27	19.07 ± 15.66	11.31 ± 7.69	24.97	0.000	0.30
**ASAT (UI/L)**	18.13 ± 6.45	12.53 ± 2.16	20.20 ± 14.10	12.60 * ± 5.69	19.86 ± 10.09	15.33 ± 8.98	21.31 ± 15.41	15.68 ± 7.94	12.85	0.001	0.18
**Alkaline Phosphatase (UI/L)**	77.06 ± 34.77	79.6 ± 21.56	61.60 ± 13.08	56.93 ± 16.69	79.60 ± 21.56	72.00 ± 20.72	59.37 ± 14.54	45.37 ± 15.51	5.62	0.02	0.09

Values are means ± SD. ETRE-PA: Early time-restricted eating combined with physical exercise group; LTRE-PA: Late time-restricted eating combined with physical exercise group; LTRE: Late time-restricted eating group; CG: Control group; LDL-c: Low-Density lipoprotein; TC: Total Cholesterol; ALAT: Alanine aminotransferase; ASAT: Aspartate aminotransferase.*: *p* < 0.05; **: *p* < 0.01; ***: *p* < 0.001.

## Data Availability

The original contributions presented in the study are included in the article, and further inquiries can be directed to the corresponding authors.

## References

[1] Okunogbe A., Nugent R., Spencer G., Powis J., Ralston J., Wilding J. (2022). Economic Impacts of Overweight and Obesity: Current and Future Estimates for 161 Countries. BMJ Glob. Health.

[2] Hales C.M., Fryar C.D., Carroll M.D., Freedman D.S., Aoki Y., Ogden C.L. (2020). Prevalence of Obesity and Severe Obesity Among Adults: United States, 2017–2018. NCHS Data Brief.

[3] McDonough D., Su X., Gao Z. (2021). Health Wearable Devices for Weight and BMI Reduction in Individuals with Overweight/Obesity and Chronic Comorbidities: Systematic Review and Network Meta-Analysis. Br. J. Sports Med..

[4] Brown J.D., Buscemi J., Milsom V., Malcolm R., O’Neil P.M. (2016). Effects on Cardiovascular Risk Factors of Weight Losses Limited to 5-10. Transl. Behav. Med..

[5] Kim J.Y. (2021). Optimal Diet Strategies for Weight Loss and Weight Loss Maintenance. J. Obes. Metab. Syndr..

[6] Lesani A., Barkhidarian B., Jafarzadeh M., Akbarzade Z., Djafarian K., Shab-Bidar S. (2023). Time-Related Meal Patterns and Breakfast Quality in a Sample of Iranian Adults. BMC Nutr..

[7] Jamshed H., Beyl R.A., Della Manna D.L., Yang E.S., Ravussin E., Peterson C.M. (2019). Early Time-Restricted Feeding Improves 24-Hour Glucose Levels and Affects Markers of the Circadian Clock, Aging, and Autophagy in Humans. Nutrients.

[8] Da Silva R.A.D., Szmuchrowski L.A., Rosa J.P.P., Santos M.A.P.d., de Mello M.T., Savoi L., Porto Y.F., de Assis Dias Martins F., Drummond M.D.M. (2023). Intermittent Fasting Promotes Weight Loss without Decreasing Performance in Taekwondo. Nutrients.

[9] James D.L., Hawley N.A., Mohr A.E., Hermer J., Ofori E., Yu F., Sears D.D. (2024). Impact of Intermittent Fasting and/or Caloric Restriction on Aging-Related Outcomes in Adults: A Scoping Review of Randomized Controlled Trials. Nutrients.

[10] Chen W., Liu X., Bao L., Yang P., Zhou H. (2023). Health Effects of the Time-Restricted Eating in Adults with Obesity: A Systematic Review and Meta-Analysis. Front. Nutr..

[11] Thomas E.A., Zaman A., Sloggett K.J., Steinke S., Grau L., Catenacci V.A., Cornier M.-A., Rynders C.A. (2022). Early Time-Restricted Eating Compared with Daily Caloric Restriction: A Randomized Trial in Adults with Obesity. Obesity.

[12] Xie Z., Sun Y., Ye Y., Hu D., Zhang H., He Z., Zhao H., Yang H., Mao Y. (2022). Randomized Controlled Trial for Time-Restricted Eating in Healthy Volunteers without Obesity. Nat. Commun..

[13] Hutchison A.T., Regmi P., Manoogian E.N.C., Fleischer J.G., Wittert G.A., Panda S., Heilbronn L.K. (2019). Time-Restricted Feeding Improves Glucose Tolerance in Men at Risk for Type 2 Diabetes: A Randomized Crossover Trial. Obesity.

[14] Kazeminasab F., Baharlooie M., Karimi B., Mokhtari K., Rosenkranz S.K., Santos H.O. (2023). Effects of Intermittent Fasting Combined with Physical Exercise on Cardiometabolic Outcomes: Systematic Review and Meta-Analysis of Clinical Studies. Nutr. Rev..

[15] Albosta M., Bakke J. (2021). Intermittent Fasting: Is There a Role in the Treatment of Diabetes? A Review of the Literature and Guide for Primary Care Physicians. Clin. Diabetes Endocrinol..

[16] Vieira R.F.L., Muñoz V.R., Junqueira R.L., de Oliveira F., Gaspar R.C., Nakandakari S.C.B.R., Costa S.d.O., Torsoni M.A., da Silva A.S.R., Cintra D.E. (2022). Time-Restricted Feeding Combined with Aerobic Exercise Training Can Prevent Weight Gain and Improve Metabolic Disorders in Mice Fed a High-Fat Diet. J. Physiol..

[17] Petridi F., Geurts J.M.W., Nyakayiru J., Schaafsma A., Schaafsma D., Meex R.C.R., Singh-Povel C.M. (2024). Effects of Early and Late Time-Restricted Feeding on Parameters of Metabolic Health: An Explorative Literature Assessment. Nutrients.

[18] Boege H.L., Bhatti M.Z., St-Onge M.-P. (2021). Circadian Rhythms and Meal Timing: Impact on Energy Balance and Body Weight. Curr. Opin. Biotechnol..

[19] BaHammam A.S., Pirzada A. (2023). Timing Matters: The Interplay between Early Mealtime, Circadian Rhythms, Gene Expression, Circadian Hormones, and Metabolism—A Narrative Review. Clocks Sleep.

[20] Sutton E.F., Beyl R., Early K.S., Cefalu W.T., Ravussin E., Peterson C.M. (2018). Early Time-Restricted Feeding Improves Insulin Sensitivity, Blood Pressure, and Oxidative Stress Even without Weight Loss in Men with Prediabetes. Cell Metab..

[21] Gabel K., Hoddy K.K., Haggerty N., Song J., Kroeger C.M., Trepanowski J.F., Panda S., Varady K.A. (2018). Effects of 8-Hour Time Restricted Feeding on Body Weight and Metabolic Disease Risk Factors in Obese Adults: A Pilot Study. Nutr. Healthy Aging.

[22] Schuppelius B., Peters B., Ottawa A., Pivovarova-Ramich O. (2021). Time Restricted Eating: A Dietary Strategy to Prevent and Treat Metabolic Disturbances. Front. Endocrinol..

[23] Maaloul R., Ben Dhia I., Marzougui H., Turki M., Kacem F.H., Makhlouf R., Amar M.B., Kallel C., Driss T., Elleuch M.H. (2023). Is Moderate-Intensity Interval Training More Tolerable than High-Intensity Interval Training in Adults with Obesity?. Biol. Sport..

[24] Batitucci G., Faria Junior E.V., Nogueira J.E., Brandão C.F.C., Abud G.F., Ortiz G.U., Marchini J.S., Freitas E.C. (2022). Impact of Intermittent Fasting Combined with High-Intensity Interval Training on Body Composition, Metabolic Biomarkers, and Physical Fitness in Women With Obesity. Front. Nutr..

[25] Beck T.W. (2013). The Importance of a Priori Sample Size Estimation in Strength and Conditioning Research. J. Strength. Cond. Res..

[26] Miladi S., Hammouda O., Ameur R., Miladi S.C., Feki W., Driss T. (2024). Time-Restricted Eating Benefits on Pulmonary Function and Postural Balance in Overweight or Obese Women. Nutrients.

[27] Brunani A., Perna S., Soranna D., Rondanelli M., Zambon A., Bertoli S., Vinci C., Capodaglio P., Lukaski H., Cancello R. (2021). Body Composition Assessment Using Bioelectrical Impedance Analysis (BIA) in a Wide Cohort of Patients Affected with Mild to Severe Obesity. Clin. Nutr..

[28] Panda S. (2016). Circadian Physiology of Metabolism. Science.

[29] Morris C.J., Yang J.N., Scheer F.A.J.L. (2012). The Impact of the Circadian Timing System on Cardiovascular and Metabolic Function. Prog. Brain Res..

[30] Garaulet M., Gómez-Abellán P., Alburquerque-Béjar J.J., Lee Y.-C., Ordovás J.M., Scheer F.A.J.L. (2013). Timing of Food Intake Predicts Weight Loss Effectiveness. Int. J. Obes..

[31] Johnston J.D., Ordovás J.M., Scheer F.A., Turek F.W. (2016). Circadian Rhythms, Metabolism, and Chrononutrition in Rodents and Humans. Adv. Nutr..

[32] Beqa Ahmeti G., Idrizovic K., Elezi A., Zenic N., Ostojic L. (2020). Endurance Training vs. Circuit Resistance Training: Effects on Lipid Profile and Anthropometric/Body Composition Status in Healthy Young Adult Women. Int. J. Environ. Res. Public Health.

[33] Friedewald W.T., Levy R.I., Fredrickson D.S. (1972). Estimation of the Concentration of Low-Density Lipoprotein Cholesterol in Plasma, without Use of the Preparative Ultracentrifuge. Clin. Chem..

[34] De Souza S.A.F., Faintuch J., Fabris S.M., Nampo F.K., Luz C., Fabio T.L., Sitta I.S., de Batista Fonseca I.C. (2009). Six-Minute Walk Test: Functional Capacity of Severely Obese before and after Bariatric Surgery. Surg. Obes. Relat. Dis..

[35] Hunter G., Seelhorst D., Snyder S. (2003). Comparison of Metabolic and Heart Rate Responses to Super Slow Vs. Traditional Resistance Training. J. Strength Cond. Res./Natl. Strength. Cond. Assoc..

[36] Macarilla C.T., Sautter N.M., Robinson Z.P., Juber M.C., Hickmott L.M., Cerminaro R.M., Benitez B., Carzoli J.P., Bazyler C.D., Zoeller R.F. (2022). Accuracy of Predicting One-Repetition Maximum from Submaximal Velocity in The Barbell Back Squat and Bench Press. J. Hum. Kinet..

[37] Crose A., Alvear A., Singroy S., Wang Q., Manoogian E., Panda S., Mashek D.G., Chow L.S. (2021). Time-Restricted Eating Improves Quality of Life Measures in Overweight Humans. Nutrients.

[38] Lin S., Cienfuegos S., Ezpeleta M., Gabel K., Pavlou V., Mulas A., Chakos K., McStay M., Wu J., Tussing-Humphreys L. (2023). Time-Restricted Eating Without Calorie Counting for Weight Loss in a Racially Diverse Population. Ann. Intern. Med..

[39] Kim J., Song Y. (2022). Early Time-Restricted Eating Reduces Weight and Improves Glycemic Response in Young Adults: A Pre-Post Single-Arm Intervention Study. Obes. Facts.

[40] Aragon A.A., Schoenfeld B.J. (2022). Does Timing Matter? A Narrative Review of Intermittent Fasting Variants and Their Effects on Bodyweight and Body Composition. Nutrients.

[41] Liu J., Yi P., Liu F. (2023). The Effect of Early Time-Restricted Eating vs Later Time-Restricted Eating on Weight Loss and Metabolic Health. J. Clin. Endocrinol. Metab..

[42] Do Nascimento Queiroz J., Macedo R.C.O., Dos Santos G.C., Munhoz S.V., Machado C.L.F., de Menezes R.L., Menzem E.N., Moritz C.E.J., Pinto R.S., Tinsley G.M. (2022). Cardiometabolic Effects of Early v. Delayed Time-Restricted Eating plus Energetic Restriction in Adults with Overweight and Obesity: An Exploratory Randomised Clinical Trial. Br. J. Nutr..

[43] Jamshed H., Steger F., Bryan D., Richman J., Warriner A., Hanick C., Martin C., Salvy S.-J., Peterson C. (2022). Effectiveness of Early Time-Restricted Eating for Weight Loss, Fat Loss, and Cardiometabolic Health in Adults with Obesity: A Randomized Clinical Trial. JAMA Intern. Med..

[44] Liu D., Huang Y., Huang C., Yang S., Wei X., Zhang P., Guo D., Lin J., Xu B., Li C. (2022). Calorie Restriction with or without Time-Restricted Eating in Weight Loss. N. Engl. J. Med..

[45] Tagawa R., Watanabe D., Ito K., Ueda K., Nakayama K., Sanbongi C., Miyachi M. (2021). Dose–Response Relationship between Protein Intake and Muscle Mass Increase: A Systematic Review and Meta-Analysis of Randomized Controlled Trials. Nutr. Rev..

[46] Young J.A., Zhu S., List E.O., Duran-Ortiz S., Slama Y., Berryman D.E. (2022). Musculoskeletal Effects of Altered GH Action. Front. Physiol..

[47] Yakupova E.I., Bocharnikov A.D., Plotnikov E.Y. (2022). Effects of Ketogenic Diet on Muscle Metabolism in Health and Disease. Nutrients.

[48] Lowe D.A., Wu N., Rohdin-Bibby L., Moore A.H., Kelly N., Liu Y.E., Philip E., Vittinghoff E., Heymsfield S.B., Olgin J.E. (2020). Effects of Time-Restricted Eating on Weight Loss and Other Metabolic Parameters in Women and Men with Overweight and Obesity: The TREAT Randomized Clinical Trial. JAMA Intern. Med..

[49] Patterson R.E., Laughlin G.A., Sears D.D., LaCroix A.Z., Marinac C., Gallo L.C., Hartman S.J., Natarajan L., Senger C.M., Martínez M.E. (2015). Intermittent Fasting and Human Metabolic Health. J. Acad. Nutr. Diet..

[50] Kotarsky C.J., Johnson N.R., Mahoney S.J., Mitchell S.L., Schimek R.L., Stastny S.N., Hackney K.J. (2021). Time-Restricted Eating and Concurrent Exercise Training Reduces Fat Mass and Increases Lean Mass in Overweight and Obese Adults. Physiol. Rep..

[51] Boyd P., O’Connor S.G., Heckman-Stoddard B.M., Sauter E.R. (2022). Time-Restricted Feeding Studies and Possible Human Benefit. JNCI Cancer Spectr..

[52] Vasim I., Majeed C.N., DeBoer M.D. (2022). Intermittent Fasting and Metabolic Health. Nutrients.

[53] Yang A., Mottillo E.P. (2020). Adipocyte Lipolysis: From Molecular Mechanisms of Regulation to Disease and Therapeutics. Biochem. J..

[54] Zhang S., Williams K.J., Verlande-Ferrero A., Chan A.P., Su G.B., Kershaw E.E., Cox J.E., Maschek J.A., Shapira S.N., Christofk H.R. (2024). Acute Activation of Adipocyte Lipolysis Reveals Dynamic Lipid Remodeling of the Hepatic Lipidome. J. Lipid Res..

[55] Schirinzi V., Poli C., Berteotti C., Leone A. (2023). Browning of Adipocytes: A Potential Therapeutic Approach to Obesity. Nutrients.

[56] Taetzsch A., Roberts S.B., Bukhari A., Lichtenstein A.H., Gilhooly C.H., Martin E., Krauss A.J., Hatch-McChesney A., Das S.K. (2021). Eating Timing: Associations with Dietary Intake and Metabolic Health. J. Acad. Nutr. Diet..

[57] Almeida G., Souza M., Pereira L. (2023). Relationship between Omitting Breakfast and Late Eating with Obesity and Metabolic Disorders: A Review Focusing on Chrononutrition. Arch. Health.

[58] Dragoo J.L., Shapiro S.A., Bradsell H., Frank R.M. (2021). The Essential Roles of Human Adipose Tissue: Metabolic, Thermoregulatory, Cellular, and Paracrine Effects. J. Cartil. Jt. Preserv..

[59] Yadav R.L., Yadav P.K., Yadav L.K., Agrawal K., Sah S.K., Islam M.N. (2017). Association between Obesity and Heart Rate Variability Indices: An Intuition toward Cardiac Autonomic Alteration—A Risk of CVD. Diabetes Metab. Syndr. Obes..

[60] Lucidi P., Perriello G., Porcellati F., Pampanelli S., Fano M., Tura A., Bolli G., Fanelli C. (2023). Diurnal Cycling of Insulin Sensitivity in Type 2 Diabetes: Evidence for Deviation from Physiology at an Early Stage. Diabetes.

[61] Lewis P., Oster H., Korf H.W., Foster R.G., Erren T.C. (2020). Food as a Circadian Time Cue-Evidence from Human Studies. Nat. Rev. Endocrinol..

[62] Manoogian E.N.C., Chow L.S., Taub P.R., Laferrère B., Panda S. (2022). Time-Restricted Eating for the Prevention and Management of Metabolic Diseases. Endocr. Rev..

[63] Ma Y.-N., Jiang X., Tang W., Song P. (2023). Influence of Intermittent Fasting on Autophagy in the Liver. Biosci. Trends.

[64] Ferrell J.M. (2023). Circadian Rhythms and Inflammatory Diseases of the Liver and Gut. Liver Res..

[65] Dai Z., Wan K., Miyashita M., Ho R.S., Zheng C., Poon E.T., Wong S.H. (2024). The Effect of Time-Restricted Eating Combined with Exercise on Body Composition and Metabolic Health: A Systematic Review and Meta-Analysis. Adv. Nutr..

[66] Real-Hohn A., Navegantes C., Ramos K., Ramos-Filho D., Cahuê F., Galina A., Salerno V.P. (2018). The Synergism of High-Intensity Intermittent Exercise and Every-Other-Day Intermittent Fasting Regimen on Energy Metabolism Adaptations Includes Hexokinase Activity and Mitochondrial Efficiency. PLoS ONE.

[67] Wei M., Brandhorst S., Shelehchi M., Mirzaei H., Cheng C.W., Budniak J., Groshen S., Mack W.J., Guen E., Di Biase S. (2017). Fasting-Mimicking Diet and Markers/Risk Factors for Aging, Diabetes, Cancer, and Cardiovascular Disease. Sci. Transl. Med..

[68] Moro T., Tinsley G., Bianco A., Marcolin G., Pacelli Q.F., Battaglia G., Palma A., Gentil P., Neri M., Paoli A. (2016). Effects of Eight Weeks of Time-Restricted Feeding (16/8) on Basal Metabolism, Maximal Strength, Body Composition, Inflammation, and Cardiovascular Risk Factors in Resistance-Trained Males. J. Transl. Med..

[69] Tinsley G.M., Moore M.L., Graybeal A.J., Paoli A., Kim Y., Gonzales J.U., Harry J.R., VanDusseldorp T.A., Kennedy D.N., Cruz M.R. (2019). Time-Restricted Feeding plus Resistance Training in Active Females: A Randomized Trial. Am. J. Clin. Nutr..

[70] Abaïdia A.-E., Daab W., Bouzid M.A. (2020). Effects of Ramadan Fasting on Physical Performance: A Systematic Review with Meta-Analysis. Sports Med..

[71] Correia J.M., Santos I., Pezarat-Correia P., Silva A.M., Mendonca G.V. (2020). Effects of Ramadan and Non-Ramadan Intermittent Fasting on Body Composition: A Systematic Review and Meta-Analysis. Front. Nutr..

[72] Perez-Montilla J.J., Cuevas-Cervera M., Gonzalez-Muñoz A., Garcia-Rios M.C., Navarro-Ledesma S. (2022). Efficacy of Nutritional Strategies on the Improvement of the Performance and Health of the Athlete: A Systematic Review. Int. J. Environ. Res. Public Health.

[73] Moro T., Tinsley G., Longo G., Grigoletto D., Bianco A., Ferraris C., Guglielmetti M., Veneto A., Tagliabue A., Marcolin G. (2020). Time-Restricted Eating Effects on Performance, Immune Function, and Body Composition in Elite Cyclists: A Randomized Controlled Trial. J. Int. Soc. Sports Nutr..

[74] Aird T.P., Farquharson A.J., Bermingham K.M., O’Sulllivan A., Drew J.E., Carson B.P. (2021). Divergent Serum Metabolomic, Skeletal Muscle Signaling, Transcriptomic, and Performance Adaptations to Fasted versus Whey Protein-Fed Sprint Interval Training. Am. J. Physiol. Endocrinol. Metab..

[75] Brady A.J., Langton H.M., Mulligan M., Egan B. (2021). Effects of 8 Wk of 16:8 Time-Restricted Eating in Male Middle- and Long-Distance Runners. Med. Sci. Sports Exerc..

[76] Kang J., Ratamess N.A., Faigenbaum A.D., Bush J.A., Beller N., Vargas A., Fardman B., Andriopoulos T. (2022). Effect of Time-Restricted Feeding on Anthropometric, Metabolic, and Fitness Parameters: A Systematic Review. J. Am. Nutr. Assoc..

[77] Tinsley G.M., Forsse J.S., Butler N.K., Paoli A., Bane A.A., La Bounty P.M., Morgan G.B., Grandjean P.W. (2017). Time-Restricted Feeding in Young Men Performing Resistance Training: A Randomized Controlled Trial. Eur. J. Sport. Sci..

[78] Correia J.M., Santos I., Pezarat-Correia P., Minderico C., Schoenfeld B.J., Mendonca G.V. (2021). Effects of Time-Restricted Feeding on Supramaximal Exercise Performance and Body Composition: A Randomized and Counterbalanced Crossover Study in Healthy Men. Int. J. Environ. Res. Public Health.

[79] Zouhal H., Bagheri R., Ashtary-Larky D., Wong A., Triki R., Hackney A.C., Laher I., Abderrahman A.B. (2020). Effects of Ramadan Intermittent Fasting on Inflammatory and Biochemical Biomarkers in Males with Obesity. Physiol. Behav..

[80] Wilk M., Zajac A., Tufano J.J. (2021). The Influence of Movement Tempo During Resistance Training on Muscular Strength and Hypertrophy Responses: A Review. Sports Med..

